# **PROTOCOL**: The effects of inclusion on academic achievement, socioemotional development and wellbeing of children with special educational needs

**DOI:** 10.1002/cl2.1170

**Published:** 2021-05-16

**Authors:** Nina T. Dalgaard, Anja Bondebjerg, Bjørn C. A. Viinholt, Trine Filges

**Affiliations:** ^1^ VIVE‐The Danish Center for Social Science Research Copenhagen Denmark

## 1 BACKGROUND

### 1.1 Description of the condition

The term *children with special educational needs* (SEN) refers to highly diverse populations of children with a wide range of physical, cognitive and socioemotional disabilities or difficulties as well as strengths and resources causing them to require varying degrees of special educational support and assistance (Squires, 2012; Vehmas, 2010; Wilson, 2002).

Several studies document significant gender imbalances in the number of students who receive special educational support, and for most disability categories the prevalence is higher for boys (Skårbrevik, 2002). The reasons for these imbalances are likely complex, and it is beyond the scope of the present review to account for the hypotheses and findings associated with each disability category. However, a general hypothesis across different categories of special educational needs is the notion that special educational needs are more likely to remain undetected in girls as symptoms and problems in girls may be less visible to educators (Arms et al., 2008).

Traditional special education consists of segregating students with special needs from mainstream students within separate and typically smaller classrooms or educational settings. However, as early as in the 1930s, a movement originally known as *mainstreaming*, and in more recent years as *inclusion*, has sought to bring an end to segregated placement as the preferred educational option for students with special needs (Carlberg & Kavale, 1980). In 1994, the idea of inclusive education became even more consolidated when the Salamanca Statement was adopted by representatives from 92 countries, resulting in an international shift in policy. This meant that far more students with special needs started entering general educational settings around the globe (Murawski & Lee Swanson, 2001; Ruijs & Peetsma, 2009).

The terms *inclusion, mainstreaming, integrated placement*, and *cross‐categorical instruction* all refer to educational settings with a group composition consisting of a mixture of students with and without special educational needs. In the present review, we have chosen to use the term *inclusion* to refer to general educational settings in which there is a mixture of students with and without special educational needs. Ideally, inclusion and inclusive education should be based on an educational approach in which the goal is to continuously address and respond to the diversity of needs of all learners through increasing participation and reducing exclusion within and from education. Inclusion thus may involve changes and modifications in content, approaches, structures and strategies, with a common vision which covers all children and a conviction that it is the responsibility of the regular system to educate all children. Inclusion emphasises the provision of opportunities for equal participation of children with disabilities (physical, social and/or emotional) whenever possible into general education, but leaves open the possibility of personal choice and options for special assistance and facilities for those who need it (UNESCO, 2005). Inclusion as an ideological and theoretical movement was built on a philosophical foundation but during the last 60 years, the number of empirical studies addressing inclusive education has grown tremendously. However, findings on the efficacy of inclusion on student outcomes are still far from unequivocal (Kavale & Forness, 2000; Lindsay, 2007; Ruijs & Peetsma, 2009). This is where the present review will contribute, as the aim of the review is to summarise contemporary evidence on the effects of inclusive education when compared to a traditional segregated approach on students' learning, socioemotional adjustment and well‐being. It is important to consider the types of students who might benefit most from inclusive education. As stated earlier, it is possible that the effects of inclusive education may be different for girls and boys. Further, a child's cognitive and socioemotional skills and needs develop throughout childhood and adolescence (Lightfoot et al., 2009) and thus, it is possible that the potential benefits of inclusive education may vary depending on the age of the children. We plan to to explore the impact of these student characteristics on student outcomes.

### 1.2 Description of the intervention

At the core of inclusion is the principle that students with special or additional learning needs or disabilities belong in mainstream education. The fundamental principle of inclusive education is that all children should learn together, regardless of any difficulties or differences (UNESCO, 2005; Winter & O'Raw, 2010). However, operationally within the present review, we define *inclusion* as an educational setting with a mixture of children with and without special educational needs. In the present review, the intervention termed *inclusion* may thus be defined as any given group composition within a general educational setting which contains at least one child with an identified special educational need.

Based on the core principles of inclusion there are many ways in which inclusion may be practised and adjusted, and thus there are a large number of characteristics within the inclusive setting, which may vary across the included studies. We will review studies of all kinds of inclusive education meaning that placement in the inclusive setting may be full time or part time. Special education students are a diverse group, as outlined in Section 4.1.2, and we will review studies regardless of the type of special educational needs of the student population and regardless of the ratio of students with and without special needs within the inclusive setting. We will include studies in which the general education teachers are provided with support and continuing professional development aimed at helping the teachers accommodate the needs of special education students and studies of inclusive settings in which no such support is offered to the teachers. It is often referred to as coteaching when two or more professionals deliver substantive instruction to a diverse or blended group of students within the same physical space (Murawski & Lee Swanson, 2001). In the present review, we will include studies, in which special education teachers and/or teaching assistants are present within the general education setting (coteaching) and studies in which they are not. For physically disabled students there may be adjustments made to the inclusive educational setting in order to accommodate aids such as wheelchairs, and for dyslexic students a number of computer programmes may be available. We will review studies in which students with special needs are provided with any kind of aid and technological support.

This list of possible variations in student and classroom characteristics is not exhaustive, and in summary, within the present review we will include studies of all kinds of inclusive education as long as the studies are aimed at exploring the effects of inclusion in comparison to segregated special educational settings. We plan to conduct moderator analyses to explore the impact of specific characteristics of the inclusive educational setting and the characteristics of special educational needs on student outcomes.

### 1.3 How the intervention might work

Considering the rapid global development towards inclusive educational placement for students with special educational needs, there is a rather surprising lack of pedagogical, psychological, or didactic theories regarding the specific ways in which inclusive education may affect students with special needs' academic and socioemotional development. Group composition within the educational setting may play a significant role in determining the academic achievement, socioemotional development and overall wellbeing of special needs students. Theoretically and ideologically, scholars favouring mainstreaming or inclusion propose that segregated educational placement causes stigmatisation and social isolation which may have detrimental effects on the self‐concept, social identity, and self‐confidence of students with special educational needs (Dyssegaard & Larsen, 2013). Second, being placed in a general education classroom along with typically developing peers is proposed to benefit special needs students' academic growth through peer effects (Rea et al., 2002; Tremblay, 2013). Finally, it is hypothesised that social interaction with general education peers may provide developmental opportunities that are not present in smaller, specialised units (Fisher & Meyer, 2002).

On the other hand, opponents of inclusive education for all special needs students suggest that placement in general education classrooms may have adverse effects for special needs children especially if the time and resources allocated for individualisation and differentiation are not aligned with student needs. In such cases, special needs students' learning opportunities and wellbeing may also suffer, resulting in damages to self‐concept (Zeleke, 2004), social isolation or bullying (Monchy et al., 2004; Pijl et al., 2010), stress (Pitt & Curtin, 2004), negative self‐perception, and lower self‐confidence (Bakker et al., 2007; Ruijs & Peetsma, 2009).

Hegarty (1993) provides a narrative review of the literature on inclusion and suggests that a number of factors are associated with positive student outcomes in inclusive settings. These are: (1) instruction based on student achievement needs, (2) materials and procedures that allow students to proceed at their own pace, (3) additional time for students who need it, (4) increased student responsibility for their own learning, (5) cooperation among students in achieving goals, (6) support teaching and (7) collaboration among special and general education teachers.

In sum, the impact of inclusion on the academic achievement, socioemotional development, and wellbeing of students with special needs may be hypothesised to be both positive and negative, and the current knowledge base is rather unclear, leaving special educators and policymakers uncertain when making decisions on special education provision.

### 1.4 Why it is important to do this review

Since the 1980s, a number of reviews on the impact of inclusion on students with special needs have been published (Madden & Slavin, 1983; Ottenbacher & Cooper, 1982; Wang & Baker, 1985; Hegarty, 1993). Results are equivocal, and several reviews point to a number of methodological challenges and weaknesses of the study designs within the included primary studies. In summary, most reviews suggest a neutral or small positive impact of inclusion on most outcomes. However, all reviews also point to the need to study the impact of potential moderators more thoroughly, as there may be several interaction effects between student and classroom characteristics, such *as student disability category* × *proportion of students with disabilities within the classroom* and *disability category* × *presence of teaching assistants*. Therefore, it is important to conduct the present review in order to explore the impact of potential moderators associated with student and classroom characteristics.

In the following section we present the existing reviews and their main findings.

In a systematic review and meta‐analysis, which included 50 primary studies exploring the effects of special versus regular class placement for children with special needs, Carlberg and Kavale (1980) concluded that for students with special needs consisting of below average IQs, special classes were significantly inferior to regular classes on all outcome measures (separate analyses were carried out for achievement, social/personal and other measures). However, for students with behavioural disorders, emotional disturbances and learning disabilities (LDs), special classes were superior to regular classes.

Madden and Slavin (1983) conducted a narrative review of the effects of mainstreaming/inclusion on students with mild academic disabilities. The review does not include a description of the search strategy for identifying records or the criteria used to determine eligibility for inclusion in the review. The review concludes that among methodologically adequate studies, findings indicate few benefits on academic and social outcomes of placement in full‐time special education compared with part‐time placement with resource support or full time regular class placement for students with mild academic disabilities.

Ottenbacher and Cooper (1982) conducted a systematic review and meta‐analysis, which included 43 primary studies exploring the effects of class placement (special class, regular class and resource class defined as placement in regular education classroom with resource support and the possibility for part time segregated education) on the social adjustment of students with mild cognitive disabilities. The overall results suggest a very small effect in favour of special class placement over regular class placement. However, when special class placement was compared with resource class placement, results were insignificant but favouring resource class placement.

Wang and Baker (1985) conducted a systematic review and meta‐analysis, which included 11 primary studies exploring the effects of mainstreaming/inclusion on children with special educational needs. In order to be eligible for inclusion in this review, primary studies needed to provide information on the effects of mainstreaming on students with special needs placed in a regular education setting. The studies had to use a control group consisting of special needs students with comparable impairment classifications placed in a segregated learning environment. The final selection of studies included 11 studies published between 1975 and 1984. The included studies used a wide variety of outcomes, but within the meta‐analysis, outcomes were synthesised into three categories: performance, attitudinal, and process effects, and separate analyses were carried out for each of the three outcome categories. The study found small‐to‐moderate beneficial effects of inclusion on all outcomes with an overall mean weighted effect size across all studies and all three categories of outcomes of 0.33.

Hegarty (1993) provides a narrative review of the literature on integration (inclusion) of students with different disabilities. The narrative review is based on a literature review which was commissioned by the Centre for Educational Research and Innovation under the Organisation for Economic Cooperation and Development (OECD) and was conducted by researchers in five different countries. The review does not include a description of the search strategies for identifying records or the criteria used to determine eligibility for the selected primary studies. Within the review, a number of factors which are associated with effective integration programmes are identified.

Baker et al. (1994/1995) describe a review and meta‐analysis by Baker et al. (1994/1995), which included 13 primary studies exploring the effects of inclusive placement on academic and social outcomes for students with special needs. We have been unable to retrieve the original publication, but according to Baker et al. (1994/1995), this study found a very small effect in favour of inclusive placement on academic outcomes (0.08) and a small to moderate effect on social outcomes (0.28).

Sebba and Sachdev (1997) provide a review as part of a research report on what works for whom in inclusive education. The review does not include a description of the search strategy or the criteria for inclusion/exclusion of studies for the review. Within the research report, the authors suggest an overall positive impact of inclusive education and list a number of potential moderators such as attitudes of teachers and parents as well as a number of recommendations for the implementation of inclusive education.

McGregor and Vogelsberg (1998) provide a narrative review of studies of both the effects of inclusive schooling on student outcomes and studies focusing on issues related to the implementation of inclusion. The review includes both quantitative and qualitative studies including case studies. Results are difficult to synthesise, but suggest an overall positive impact of inclusion based on the main findings: (1) students with disabilities demonstrate high levels of social interaction in settings with typically developing peers, but placement alone does not guarantee positive social outcomes; (2) interactive small group contexts facilitate skill acquisition and social acceptance; (3) friendships develop between students with disabilities and typically developing peers.

Freeman and Alkin (2000) conducted a systematic narrative review in which it was concluded that on measures of academic achievement and social competence, children with mental retardation placed in general education perform better than children with mental retardation placed in special education classrooms. This review was only about children with mental retardation and did not include meta‐analyses.

Murawski and Lee Swanson (2001) conducted a systematic review and meta‐analysis which included 6 studies exploring the effectiveness of coteaching on student outcomes of both general education students and students with special educational needs. Coteaching was defined as two or more professionals delivering substantive instruction to a diverse or blended group of students within a shared/common physical space, and thus in this review coteaching is a form of inclusion. The outcomes within the included studies were grades, achievement scores, social and attitudinal outcomes. The review found coteaching to be effective (average total effect size of 0.40). It is unclear what the control conditions within the included studies were and two of the included studies did not have a control group, but used a pre‐test/post‐test research design.

Lindsay (2007) provides a narrative review of the effectiveness of inclusive education for students with special educational needs. The review provides a historical overview of the vast literature prior to 2000 and a search of studies published 2001–2005 in eight journals on special education. The search identified 1373 studies and points to the fact that only 1% of the identified papers were comparative outcome studies. The review concludes that there is a lack of evidence for the effectiveness of inclusion and argues that where evidence does exist, the balance is only marginally positive. Lindsay (2007) thus supports the need for an updated systematic review and meta‐analysis on the effectiveness of inclusion for students with special needs, with special attention to the potential impact of student and classroom moderators.

In a systematic narrative review of the effects of inclusion on both learning and socioemotional outcomes of students with and without special needs, Ruijs and Peetsma (2009) point to mixed findings regarding the effects of inclusion on student outcomes and suggest a number of potential moderators. The authors conclude that there is a need for more research. This review has not been updated since publication and does not include meta‐analyses.

In 2009, a systematic review of evidence comparing the academic performance of students with special needs in different educational settings was carried out by the Canadian Council on Learning. The review included 30 primary studies. The search strategy for identifying studies was not described. The included studies examined students with LDs, intellectual disabilities, language impairments and mixed disabilities. The quality of each study was rated as either “high”, “medium”, or “low” based on criteria related to transparency and research design, and effect sizes were retrieved. No meta‐analyses were carried out, but the authors provide tables illustrating the number of effect sizes for each disability category favouring either inclusive or segregated settings along with the quality ratings of the studies from which they were retrieved. The authors point to mixed findings but conclude that the balance of evidence shows favourable academic outcomes for students with special educational needs educated in inclusive settings, however they also note that results are not homogenous and that effects are generally small in magnitude.^1^

Dyssegaard and Larsen (2013) provide a systematic review and narrative synthesis on the effects of including children with special needs in mainstream teaching in primary and lower secondary school, and on which of the applied educational methods have proven to have a positive effect. The narrative synthesis is based on 43 studies of which 16 studies were deemed to have a “high level of evidence”. The included studies consist of randomised controlled trials, non‐randomised controlled trials, systematic reviews, cohort studies, longitudinal studies, and studies using a pre‐test/post‐test design. The systematic review included studies focusing on outcomes for both mainstream and special needs students and does not include a meta‐analysis. The conclusion points to mixed findings regarding the overall effectiveness of inclusion on the academic achievement and psychosocial adjustment of special needs students and suggests that the effects may vary depending on the age of the child and the overall school and teacher attitudes towards inclusion. Furthermore, the review suggests that the effectiveness of coteaching may depend on the educational background and continuous professional development of both special and general education teachers and of teaching assistants.

Carroll et al. (2017) provide a rapid evidence assessment of studies focused on approaches, strategies, and interventions supporting children and young people with special educational needs in mainstream schools. The rapid evidence assessment is based on a systematic search in a single database (ERIC) as well as a strategy of consulting experts within the relevant fields. The initial search identified 1046 papers of which 505 were later excluded due to low quality of evidence. The rapid evidence assessment points to a number of implementation strategies, pedagogical and didactic approaches which have shown positive results. Furthermore, the study points to evidence gaps and suggests the need for further research. The rapid evidence assessment does not include a meta‐analysis.

In the present review, besides being up to date, we will conduct an extensive risk of bias assessment of all included studies, and we will provide separate meta‐analyses for each conceptual outcome (academic achievement, socioemotional development and wellbeing). Furthermore, we hope to be able to conduct moderator analyses based on the children's specific disability categories and the specific type of inclusion setting. This may shed further light on the initial differential findings from existing reviews.

Traditional segregated special education is costly and in a time of increased interaction between special and general education systems and constraints on education spending, policymakers must consider the cost‐efficiency of different special needs provisions.

As more students with special educational needs enter general education settings, educators and policymakers must consider how the needs of these students are met in different settings and on what grounds placement in general or special educational settings should be determined. As previously noted, the current knowledge base is ambiguous with many findings suggesting a complex interplay between student and classroom characteristics (Carlberg & Kavale, 1980; Mesibov & Shea, 1996; Peetsma et al., 2001), leaving special educators and policymakers uncertain when making decisions on special education provision and highlighting the need for a comprehensive review of the effectiveness of *inclusion* on student outcomes.

## 2 OBJECTIVES

The objective of this systematic review is firstly:

To uncover and synthesise data from studies to assess the effects of inclusion on measures of academic achievement, socioemotional development and wellbeing of children with special needs when compared to children with special needs who receive special education in a segregated setting.

A secondary objective is to explore how potential moderators (gender, age, type of special need, part or full time inclusive education, and coteaching) affect the outcomes. The moderator analysis will be performed as outlined in Section 4.3.11.

## 3 METHODS

### 3.1 Criteria for considering studies for this review

#### 3.1.1 Types of studies

In order to summarise what is known about the causal effects of inclusion on student's academic achievement, socioemotional outcomes, and wellbeing in special education, we will include all studies with a well‐defined control group. Thus, the study designs eligible for inclusion are:

A. Randomised and quasi‐randomised controlled trials (allocated at either the individual level or cluster level, e.g., class/school/geographical area etc.).
B. Non‐randomised studies (inclusion has occurred in the course of usual decisions, the allocation to inclusive and segregated special educational placement is not controlled by the researcher, and there is a comparison of two or more groups of participants, i.e., at least a treated group and a control group).

Studies using a single group pretest/posttest research design will not be eligible for inclusion in the review. Non‐randomised studies using an instrumental variable approach will not be included—see the Appendix C (*Justification of exclusion of studies using an instrumental variable (IV) approach*) for our rationale for excluding studies of these designs.

In order to minimise the risk of bias in cluster randomised studies, we will exclude study designs in which only one unit was assigned to the intervention or control group. That is, there must be at least two units in the intervention group and two units in the control group, as there is otherwise a substantial risk of confounding treatment effects with “unit” effects (in this case, “unit” would likely be school).

In order to maximise the relevance of findings from the present review to current policy and decision makers, we will limit our search to studies published after 2000. The reason for excluding older studies is two‐fold. First, as described previously, a number of systematic reviews and meta‐analyses have already synthesised the effects of inclusion based on studies published prior to 2000. Second, educational settings, pedagogical approaches and the development and availability of technological tools to support the educational needs of special needs children have undergone major changes throughout the past two decades (Cheng & Lai, 2019), and in order for the findings from the present review to be applicable to the current realities within educational settings, we will limit our review to the more recent findings.

#### 3.1.2 Types of participants

The review will include special needs children in grades K to 12 (or the equivalent in European countries) in special education in the Western world defined as the OECD countries. The reasons for focusing on the OECD countries are twofold, first, we believe that the way in which children with disabilities are perceived within society is culturally embedded, which creates fundamental differences in the life circumstances for children living with disabilities around the globe (Maloni et al., 2010; McNally & Mannan, 2013). Second, special education is costly and thus the resources available for providing special educational support for children with special needs are often fundamentally different between countries in the OECD and the developing countries (Sibanda, 2018; UNESCO, 2019).

Some controversy exists regarding the definition of what constitutes a special educational need (Vehmas, 2010, Wilson, 2002). A widely used definition can be found in the US Individuals with Disabilities Education Act (IDEA), in which special needs are divided into 13 different disability categories under which children are eligible for services.^2^ These categories are:

- Specific LD (covers challenges related to a child's ability to read, write, listen, speak or do math, e.g., dyslexia or dyscalculia),
- Other health impairment (covers conditions limiting a child's strength, energy or alertness, e.g., ADHD),
- Autism spectrum disorder (ASD),
- Emotional disturbance (may include, e.g., anxiety, obsessive‐compulsive disorder and depression),
- Speech or language impairment (covers difficulties with speech or language, e.g., language problems affecting a child's ability to understand words or express herself),
- Visual impairment (covers eyesight problems, including partial sight and blindness),
- Deafness (covers instances where a child cannot hear most or all sounds, even with a hearing aid),
- Hearing impairment (refers to a hearing loss not covered by the definition of deafness),
- Deaf‐blindness (covers children suffering from both severe hearing and vision loss),
- Orthopaedic impairment (covers instances when a child has problems with bodily function or ability, as in the case of cerebral palsy),
- Intellectual disability (covers below‐average intellectual ability),
- Traumatic brain injury (covers brain injuries caused by accidents or other kinds of physical force),
- Multiple disabilities (children with more than one condition covered by the IDEA criteria).

However, the above listed criteria are not to be conceived as exhaustive or as clear‐cut definitions of what constitutes special educational needs but are rather seen as guidance tools in the search for and screening of relevant studies. We acknowledge that existing attempts to define special educational needs, as discussed in Vehmas (2010) and Wilson (2002), are characterised by a lack of clarity, which requires us to be transparent as to our own use of the term throughout the review process. For the purpose of this review we will include studies of all types of verifiable special needs, that is children who receive special educational support and/or who have been diagnosed with any kind of disability.

#### 3.1.3 Types of interventions

*Inclusion* refers to an educational setting with a mixture of children with and without special educational needs. In the present review, the intervention termed *inclusion* may thus be defined as any given group composition within a general educational setting which contains at least one child with an identified special educational need. Within some studies, inclusion may also be referred to as *integration, mainstreaming, integrated placement*, and *coteaching with a blended student population*.

Inclusion may be full‐time or part‐time and may involve additional teaching and/or pedagogical resources. We will include studies of all kinds of inclusive education.

#### 3.1.4 Types of outcome measures

In the present review, we will extract the following types of outcomes:

##### Academic achievement

Academic achievement outcomes include reading and mathematics as well as measures of other academic subjects and global academic performance. Outcome measures must be standardised measures of academic achievement such as standardised literacy tests (e.g., reading, spelling, and writing) and standardised numeracy tests (e.g., mathematical problem‐solving, arithmetic and numerical reasoning, grade level math), standardised tests in other academic subjects (e.g., in science or second language). Examples of measures of global academic performance which may be included in the review are:

- Woodcock‐Johnson III Tests of Achievement (Mather et al., 2001),
- Stanford Achievement Test (SAT) (The Psychological Corporation, 1990),
- Grade Point Average.

##### Socioemotional outcomes

Socioemotional outcomes refer to validated measures of children's psychological, emotional and social adjustment, and mental health. Examples of relevant measures which may be included are:

- The Strengths and Difficulties Questionnaire (SDQ) (Goodman, 2001),
- The Child Behaviour Checklist (CBCL) (Achenbach & Ruffle, 2000),
- The development and well‐being assessment (DAWBA) (Goodman et al., 2000).

##### Wellbeing

Wellbeing refers to measures of children's subjective quality of life, self‐perception, self‐esteem, and self‐image. Examples of relevant measures which may be included in the review are:

- The Perceived Competence Scale for Children (Harter, 1982),
- The Loneliness Scale (Asher et al., 1984),
- The Kidscreen questionnaires (Europe, 2006),
- The Self‐Esteem Index (Brown & Alexander, 1991).

Any adverse effects will be reported.

Studies who do not report on any of the outcomes listed above will be excluded from the review.

##### Primary outcomes

Academic achievement, socioemotional outcomes, and wellbeing are all primary outcomes.

##### Secondary outcomes

In addition to the primary outcomes, we will consider school completion rates as a secondary outcome.

###### Duration of follow‐up

We will include post‐intervention outcomes measured during and after placement in an inclusive educational setting. Follow‐up at any given point in time will be included. We will include follow‐up data regarding children's development and well‐being throughout the children's life course. If we include follow‐up data, separate meta‐analyses will be carried out by grouping included time points in meaningful intervals such as (1) 0–1 year follow up, (2) 1–2 year follow up, and (3) more than 2 year follow up.

###### Types of settings

In this review, we will include studies of special needs children placed in any general education setting. We will exclude children in home‐ or preschool.

### 3.2 Search methods for identification of studies

#### 3.2.1 Search strategy

Relevant studies will be identified through searches in electronic databases, governmental and grey literature repositories, hand search in specific targeted journals, citation tracking, contact to international experts and internet search engines.

#### 3.2.2 Electronic searches

##### Electronic databases

The following electronic databases will be searched:

- ERIC (EBSCO)
- Academic Search (EBSCO)
- EconLit (EBSCO)
- PsycINFO (EBSCO)
- SocIndex (EBSCO)
- International Bibliography of the Social Sciences (ProQuest)
- Sociological Abstracts (ProQuest)
- Science Citation Index Expanded (Web Of Science)
- Social Sciences Citation Index (Web Of Science)

###### Description of search‐string

The search string is based on the PICO(s)‐model, and contains three concepts, of which we have developed three corresponding search facets: population, intervention, and study type/methodology. The search string includes searches in title, abstract and subject terms for each facet. The subject terms in the facets will be chosen accordingly to each databases options.

###### Example of a search string

The search string below from the ERIC database exemplifies the search as it will be performed. The searches are structured in the following order:

- Search 1–4 covers the population
- Search 5–8 covers the intervention
- Search 9–12 covers the study types/methodology

These three facets are combined in the final search on each database (S12 in the example). The search fields covering the subject terms (S3, S7 and S11) will be modified accordingly to each database.

**Table:** 

S13	S4 AND S8 AND S12
S12	S9 OR S10 OR S11
S11	DE “Effect Size” OR DE “Control Groups” OR DE “Experimental Groups” OR DE “Experiments” OR DE “Matched Groups” OR DE “Quasiexperimental Design” OR DE “Randomized Controlled Trials” OR DE “Randomised Controlled Trials” OR DE “Comparative Testing”
S10	AB (effect* OR trial* OR experiment* OR control* OR random* OR impact* OR compar* OR difference*)
S9	TI (effect* OR trial* OR experiment* OR control* OR random* OR impact* OR compar* OR difference*)
S8	S5 OR S6 OR S7
S7	DE “Placement” OR DE “Academic Accommodations (Disabilities)” OR DE “Inclusion” OR DE “Mainstreaming” OR DE “Student Placement”
S6	AB (integrat* OR immers* OR inclus* OR mainstream* OR placement*)
S5	TI (integrat* OR immers* OR inclus* OR mainstream* OR placement*)
S4	(S1 AND S2) OR S3
S3	DE (“Special Needs Students”)
S2	TI (need* OR special* OR additional*) OR AB ((special* OR additional* OR educational*) N5 (need*))
S1	TI (student* OR pupil* OR child* OR youth* OR young*) OR AB (student* OR pupil* OR child* OR youth* OR young*)

###### Limitations of the search‐string

Searches will be limited to from 01/01/2000 and forwards. We will not implement any language restrictions to our search.

#### 3.2.3 Searching other resources

##### Searching other resources

###### Hand‐search

We will conduct a hand search of the following journals, in order to make sure that all relevant articles are found. The hand search will focus on editions published between 2016 and 2020 in order to secure recently unpublished articles which have not yet been indexed in the bibliographic databases. A number of specific journals will be hand‐searched. We will decide upon which journals to hand search based on the identified records from the electronic searches. The following are examples of specific journals which we may decide to hand search:

- International Journal of Inclusive Education
- British Journal of Special Education (BJSE)
- European Journal of Special Needs Education
- Journal of Research in Special Educational Needs (JORSEN)
- Journal of Intellectual Disability Research
- Disability Studies Quarterly
- Intellectual and Developmental Disabilities
- Disability, Development and Education
- Mental Retardation
- Journal of Learning Disabilities
- Exceptional Children
- British Journal of Educational Psychology
- Teacher Education and Special Education
- International Journal of Educational Management

###### Searches for unpublished literature

Most of the resources searched for unpublished literature includes multiple types of references. As an example, the resources listed to identify reports from national bibliographical resources also include working papers and dissertations, as well as peer‐reviewed references.

For the sake of transparency, we have split the resources for each type of unpublished literature. But in general, there is a great amount of overlap between the types of references in the chosen resources. The resources are listed once under the category of literature we expect to be most prevalent in the resource, even though multiple types of unpublished/published literature might be identified in the resource.

Due to the language skills of the review team, we have selected to search for additional unpublished literature in Danish, Swedish and Norwegian (other languages).

We will implement a simplified version of the search string on the resources where searches through a search‐field is possible. The simplified search will consist of intervention terms and either population or study type terms. The searches performed will be listed in the final reviews search reporting section.

*Searches for dissertations and theses in English*

- ProQuest Dissertations & Theses Global (ProQuest)
*Searches for working papers and conference proceedings in English*
- Open Grey—http://www.opengrey.eu/
- Google Scholar—https://scholar.google.com/
- Social Science Research Network—https://www.ssrn.com/index.cfm/en/
- OECD iLibrary—https://www.oecd-ilibrary.org/
- NBER working paper series—http://www.nber.org
- European Educational Research Association (EERA)—https://eera-ecer.de/
- American Educational Research Association (AERA)—https://www.aera.net/

###### Search for reports and on‐going studies in English

- Google searches—https://www.google.com/
- Best Evidence Encyclopedia—http://www.bestevidence.org/
- Social Care Online—https://www.scie-socialcareonline.org.uk/

###### Searches for dissertations, theses, working papers and proceedings in other languages

- Forskning.ku—Academic publications from the university of Copenhagen—https://forskning.ku.dk/soeg/
- AAU Publications—Academic publications from the University of Aarhus—https://pure.au.dk/portal/da/organisations/8000/publications.html
- SwePub—Academic publications at Swedish universities—http://swepub.kb.se/
- NORA—Norwegian Open Research Archives—http://nora.openaccess.no/
- DIVA—Swedish Digital Scientific Archives—http://www.diva-portal.org/smash/
- Skolporten—Swedish Dissertations—https://www.skolporten.se/forskning/

###### Search for systematic reviews

Prior to this protocol, we developed a specific search string to identify other systematic reviews in the databases listed above. This was done simultaneously with the development of the search‐string described above, and the identified relevant reviews are considered in this protocol.

We will also search for further systematic reviews on the following resources:

- Campbell Journal of Systematic Reviews—https://campbellcollaboration.org/
- Cochrane Library—https://www.cochranelibrary.com/
- Centre for Reviews and Dissemination Databases—https://www.crd.york.ac.uk/CRDWeb/
- EPPI‐Centre database of education research—https://eppi.ioe.ac.uk/webdatabases/Intro.aspx?ID=6

###### Citation‐tracking and snowballing methods of systematic reviews

Systematic reviews identified during the search process will be citation tracked in order to identify additional relevant references. Furthermore, we will utilise forwards citation‐tracking methods on key systematic reviews. The systematic reviews selected for citation tracking will be listed in the search reporting section of the systematic review.

###### Citation‐tracking and snowballing methods of individual references

We will select the most recently published, and the most cited key references for citation tracking. We will select studies from the pool of included references after the title/abstract screening is finished. The number of key references we will select is subject to change, but we expect to select approximately 20 (10 recent, 10 most cited). The studies selected for citation tracking/snowballing will be listed in the search reporting section of the systematic review.

###### Contact to experts

We will contact international experts to identify unpublished and ongoing studies, and provide them with the inclusion criteria for the review along with the list of included studies, asking for any other published, unpublished or ongoing studies relevant for the review. We will primarily contact corresponding authors of the related reviews mentioned in the section Prior reviews, but extend the contacts to others if we find references to or mentions of ongoing studies in screened publications.

### 3.3 Data collection and analysis

#### 3.3.1 Description of methods used in primary research

Based on the existing reviews, we expect to find a very limited number of RCTs, and thus we expect that the majority of relevant studies will be quasi‐experimental. An example of a study which may be included in the review is Tremblay (2013). This quasi‐experimental study compared two instructional models for students with LDs. The first model consisted of inclusion with coteaching and the second consisted of solo‐taught special education. The study used matched comparison groups. The total sample consisted of 353 students: of these, 58 students had LDs and were placed in a regular class, and 100 students had learning difficulties and were placed in a special education class. The remaining participants were students without LDs. The study used academic tests and grades as outcome measures. The study found significant differences in student outcomes in reading/writing and attendance in favour of the inclusive setting. For the purpose of the present review, we would only extract effects for the students with special educational needs. Another example of a study, which may be included in the review, is the quasi‐experimental study by Cole et al. (2004). This study compared the academic progress of students with mild LDs (*N* = 429) across inclusive and noninclusive settings in grades 2–5 in 23 schools during the course of one school year across the state of Indiana in the US. The academic progress of students was measured using a curriculum‐based measure: The Basic Academic Skills Sample (BASS). For students with disabilities, the study found no significant differences in academic progress between inclusive and non‐inclusive settings.

#### 3.3.2 Selection of studies

Under the supervision of review authors, two review team assistants will first independently screen titles and abstracts to exclude studies that are clearly irrelevant. Studies considered eligible by at least one assistant or studies where there is insufficient information in the title and abstract to judge eligibility, will be retrieved in full text. The full texts will then be screened independently by two review team assistants under the supervision of the review authors. Any disagreement of eligibility will be resolved by the review authors. Exclusion of studies that otherwise might be expected to be eligible will be documented and presented in an appendix.

The study inclusion criteria will be piloted by the review authors (see Appendix A part X, “First and second level screening”). The overall search and screening process will be illustrated in a flow diagram. None of the review authors will be blind to the authors, institutions, or the journals responsible for the publication of the articles.

#### 3.3.3 Data extraction and management

Two review authors will independently code and extract data from included studies. A coding sheet will be piloted on several studies and revised as necessary (see Appendix B). Disagreements will be resolved by consulting a third review author with extensive content and methods expertise. Disagreements resolved by a third reviewer will be reported. Data and information will be extracted on: available characteristics of participants, intervention characteristics and control conditions, research design, sample size, risk of bias and potential confounding factors, outcomes, and results. Extracted data will be stored electronically.

#### 3.3.4 Assessment of risk of bias in included studies

##### Assessment of risk of bias in included studies

We will assess the risk of bias in randomised studies using Cochrane's revised risk of bias tool, ROB 2 (Higgins et al., 2019).

The tool is structured into five domains, each with a set of signalling questions to be answered for a specific outcome. The five domains cover all types of bias that can affect results of randomised trials.

The five domains for individually randomised trials are:

(1) Bias arising from the randomisation process;
(2) Bias due to deviations from intended interventions (separate signalling questions for effect of assignment and adhering to intervention);
(3) Bias due to missing outcome data;
(4) Bias in measurement of the outcome;
(5) Bias in selection of the reported result.
  For cluster‐randomised trials, an additional domain is included ((1b) Bias arising from identification or recruitment of individual participants within clusters). We will use the latest template for completion (currently it is the version of 15 March 2019 for individually randomised parallel‐group trials and 20 October 2016 for cluster randomised parallel‐group trials). In the cluster randomised template however, only the risk of bias due to deviation from the intended intervention (effect of assignment to intervention; intention to treat ITT) is present and the signalling question concerning the appropriateness of the analysis used to estimate the effect is missing. Therefore, for cluster randomised trials we will only use the signalling questions concerning the bias arising from identification or recruitment of individual participants within clusters from the template for cluster randomised parallel‐group trials; otherwise we will use the template and signalling questions for individually randomised parallel‐group trials.
  We will assess the risk of bias in nonrandomised studies, using the model ROBINS‐I, developed by members of the Cochrane Bias Methods Group and the Cochrane Non‐Randomised Studies Methods Group (Sterne et al., 2016). We will use the latest template for completion (currently it is the version of 19 September 2016).
  The ROBINS‐I tool is based on the Cochrane RoB tool for randomised trials, which was launched in 2008 and modified in 2011 (Higgins et al., 2011).
  The ROBINS‐I tool covers seven domains (each with a set of signalling questions to be answered for a specific outcome) through which bias might be introduced into nonrandomised studies:
  (1) Bias due to confounding;
  (2) Bias in selection of participants;
  (3) Bias in classification of interventions;
  (4) Bias due to deviations from intended interventions;
  (5) Bias due to missing outcome data;
  (6) Bias in measurement of the outcome;
  (7) Bias in selection of the reported result.

The first two domains address issues before the start of the interventions and the third domain addresses classification of the interventions themselves. The last four domains address issues after the start of interventions and there is substantial overlap for these four domains between bias in randomised studies and bias in nonrandomised studies trials (although signalling questions are somewhat different in several places, see Sterne et al., 2016, and Higgins et al., 2019).

Randomised study outcomes are rated on a “Low/Some concerns/High” scale on each domain; whereas nonrandomised study outcomes are rated on a “Low/Moderate/Serious/Critical/No Information” scale on each domain. The level “Critical” means: the study (outcome) is too problematic in this domain to provide any useful evidence on the effects of intervention and it is excluded from the data synthesis. The same critical level of risk of bias (excluding the result from the data synthesis) is not directly present in the RoB 2 tool, according to the guidance to the tool (Higgins et al., 2019).

In the case of a RCT, where there is evidence that the randomisation has gone wrong or is no longer valid, we will assess the risk of bias of the outcome measures using ROBINS‐I instead of ROB 2. Examples of reasons for assessing RCTs using the ROBINS‐I tool may include studies showing large and systematic differences between treatment conditions while not explaining the randomisation procedure adequately suggesting that there was a problem with the randomisation process; studies with large scale differential attrition between conditions in the sample used to estimate the effects; or studies selectively reporting results for some part of the sample or for only some of the measured outcomes. In such cases, differences between the treatment and control conditions are likely systematically related to other factors than the intervention and the random assignment is, on its own, unlikely to produce unbiased estimates of the intervention effects. Therefore, as ROBINS‐I allow for an assessment of for example confounding, we believe it is more appropriate to assess effect sizes from studies with a compromised randomisation using ROBINS‐I than ROB 2. If so, we will report this decision as part of the risk of bias assessment of the outcome measure in question. As other effect sizes assessed with ROBINS‐I, these effect sizes may receive a ‘Critical’ rating and thus be excluded from the data synthesis.

We will stop the assessment of a nonrandomised study outcome as soon as one domain in the ROBINS‐I is judged as ‘Critical’.

‘Serious’ risk of bias in multiple domains in the ROBINS‐I assessment tool may lead to a decision of an overall judgement of ‘Critical’ risk of bias for that outcome and it will be excluded from the data synthesis.

###### Confounding

An important part of the risk of bias assessment of non‐randomised studies is consideration of how the studies deal with confounding factors. Systematic baseline differences between groups can compromise comparability between groups. Baseline differences can be observable (e.g., age and gender) and unobservable (to the researcher; e.g., children's motivation and ‘ability’). There is no single non‐randomised study design that always solves the selection problem. Different designs represent different approaches to dealing with selection problems under different assumptions, and consequently require different types of data. There can be particularly great variations in how different designs deal with selection on unobservables. The “adequate” method depends on the model generating participation, THAT IS, assumptions about the nature of the process by which participants are selected into a programme.

A major difficulty in estimating causal effects of inclusive education is the heterogeneity of children with special educational needs. In addition to the pre‐specified confounding factors, there may be unobservable factors affecting child development and well‐being or invisible selection mechanisms causing certain types of families to choose a specific educational setting for their child for reasons unavailable to the researcher.

As there is no universally correct way to construct counterfactuals for non‐randomised designs, we will look for evidence that identification is achieved, and that the authors of the primary studies justify their choice of method in a convincing manner by discussing the assumption(s) leading to identification (the assumption(s) that make it possible to identify the counterfactual). Preferably, the authors should make an effort to justify their choice of method and convince the reader that the special needs students in inclusive versus segregated settings are comparable. The judgement is reflected in the assessment of the confounder unobservables in the list of confounders considered important at the outset (see Appendix A *User guide for unobservables*).

In addition to unobservables, we have identified the following observable confounding factors to be most relevant: performance at baseline, age/gender of the child, special needs category and impairment level, and socioeconomic background of the child's family. In each study, we will assess whether these factors have been considered, and in addition we will assess other factors likely to be a source of confounding within the individual included studies.

###### Importance of pre‐specified confounding factors

The motivation for focusing on performance at baseline, age/gender of the child, special needs category and impairment level, and the socioeconomic background of the child's family is given below.

Performance at baseline is perhaps the most important potential confounding factor, as students with special needs constitute a highly diverse population. Thus we will look for evidence that students in both intervention and control group had similar academic performance at baseline.

The younger the child, the more dependent the child is on stimulating adult/child interaction. Therefore, the impact of inclusive versus segregated special education may vary depending on the age of the children, with younger children perhaps benefiting more from placement in smaller specialised units with a lower student/teacher ratio, meaning a lower number of students per teacher. Furthermore, puberty may bring about additional challenges for special education students, which may make them more socially and psychologically vulnerable to the stigma associated with having a special educational need, and it is unclear if the potential social and psychological vulnerability is best handled within a general or special educational setting. In any case, it is highly possible that the effects of inclusion may vary depending on the age of the child.

From a very early age, gender is associated with differences in child behaviour and cognition (Chaplin & Aldao, 2013; Ostrov & Keating, 2004; Silverman, 2003). Little girls and boys often show different toy and play preferences (Todd et al., 2017), and a number of studies suggest that for diagnoses such as autism spectrum disorders and ADHD there are significant gender differences in the behavioural expressions of symptoms (Halladay et al., 2015), and thus it is possible that gender may have an impact on what constitutes the best educational setting for special needs students.

As can be seen in the definition of special educational needs, the disability categories cover a very broad range of disabilities and there may be considerable variance in the impairment levels of students between the different disability categories. In the existing reviews, some results suggest that the effects of inclusive versus segregated placement may vary depending on the particular special education student population (see for instance Carlberg & Kavale, 1980, or the 2009 review from the Canadian Council of Education), which is why we consider this an important potential confounder.

A large body of research documents the impact of parental socioeconomic background on almost all aspects of children's development (Sigel & Renninger, 2006), which is why we also consider it important to control for this.

###### Effect of primary interest and important cointerventions

We are mainly interested in the effect of starting and adhering to the intended intervention, that is, the treatment on the treated (TOT) effect or students enroled in and attending inclusive education. The risk of bias assessments will therefore be in relation to this specific effect. The risk of bias assessments of both randomised trials and nonrandomised studies will consider adherence and differences in additional interventions (“co‐interventions”) between intervention groups.

Important cointerventions we will consider are interventions performed in school, during the regular school year, which are complementary to regular classes and school activities such as tutoring, short‐term reading or math interventions, or socioemotional support groups for students with a specific disability. Furthermore we will consider technological tools available to students with special educational needs as important cointerventions. Cointerventions may be delivered individually, in class, or in group sessions.

###### Assessment

At least two review authors will independently assess the risk of bias for each relevant outcome from the included studies. Any disagreements will be resolved by a third reviewer with content and statistical expertise and will be reported. We will report the risk of bias assessment in risk of bias tables for each included study outcome in the completed review.

#### 3.3.5 Measures of treatment effect

##### Continuous outcomes

For continuous outcomes, effects sizes with 95% confidence intervals will be calculated, where means and standard deviations are available. If means and standard deviations are not available, we will calculate SMDs from *F* ratios, *t* values, *χ* ^2^ values, and correlation coefficients, where available, using the methods suggested by Lipsey and Wilson (2001). If not enough information is yielded, the review authors will request this information from the principal investigators. Hedges' *g* will be used for estimating standardised mean differences (SMD). Any standardised measures of student academic achievement (e.g., reading and math), are examples of relevant continuous outcomes in this review.

##### Dichotomous outcomes

For dichotomous outcomes, we will calculate odds ratios with 95% confidence intervals. Children who pass or fail an exam is an example of a relevant dichotomous outcome in this review. There are statistical approaches available to re‐express dichotomous and continuous data to be pooled together (Sánchez‐Meca et al., 2003). In order to calculate common metric odds ratios will be converted to SMD's effect sizes using the Cox transformation. We will only transform dichotomous effect sizes to SMD's if appropriate, as may be the case with the outcomes measuring behaviour problems or psychosocial adjustment, which can be measured with binary data based on clinical cut‐offs and with continuous data.

When effect sizes cannot be pooled, study‐level effects will be reported in as much detail as possible. Software for storing data and statistical analyses will be RevMan 5.4, Excel, R and Stata 16.

#### 3.3.6 Unit of analysis issues

We will take into account the unit of analysis of the studies to determine whether individuals were randomised in groups (i.e., cluster‐randomised trials), whether individuals may have undergone multiple interventions, whether there were multiple treatment groups, and whether several studies are based on the same data source.

##### Clustered assignment of treatment

Errors in statistical analysis can occur when the unit of allocation differs from the unit of analysis. In cluster randomised trials, participants are randomised to treatment and control groups in clusters, either when data from multiple participants in a setting are included (creating a cluster within the school or community setting), or when participants are randomised by treatment locality or school. Non‐randomised studies may also include clustered assignment of treatment. Effect sizes and standard errors from such studies may be biased if the unit‐of‐analysis is the individual and an appropriate cluster adjustment is not used (Higgins & Green, 2011).

If possible, we will adjust effect sizes individually using the methods suggested by Hedges (2007) and information about the intra‐cluster correlation coefficient (ICC), realised cluster sizes, and/or estimates of the within and between variances of clusters. If it is not possible to obtain this information, we will adjust effect sizes using estimates from the literature of the ICC (e.g., Hedges & Hedberg, 2007), and assume equal cluster sizes.

We will use the ICC from the pretest covariate models for the most relevant grades, outcome measures and demography of the population under investigation. Tables 2–7 in Hedges and Hedberg (2007) offers estimates of ICC separately for grades K‐12 for mathematics achievement and reading achievement respectively and for all schools (in their sample), low–socioeconomic status schools and low‐achievement schools respectively. We will further test the sensitivity of the results for different levels of the ICC.

To calculate an average cluster size, we will divide the total sample size in a study by the number of clusters (typically the number of classrooms or schools).

##### Mixed student population

Some studies may report outcomes for a population of students consisting of both children with and without special educational needs. In the data synthesis we will only use studies in which it is possible to extract a separate effect size for children with special needs. If this information is not available in the included reports, we will attempt to contact authors to ask for separate effect size estimates. If this is unsuccessful, the study will be included in the review and reported in as much detail as possible, but will not be used in the data synthesis.

##### Multiple intervention groups and multiple interventions per individual

Studies with multiple intervention groups with different individuals will be included in this review, although only intervention and control groups that meet the eligibility criteria will be used in the data synthesis. To avoid problems with dependence between effect sizes we will apply robust standard errors (Hedges et al., 2010) and use the small sample adjustment to the estimator itself (Tipton, 2015). We will use the results in Tanner‐Smith and Tipton (2014) and Tipton (2015) to evaluate if there are enough studies for this method to consistently estimate the standard errors. See Section 4.3.10 below for more details about the data synthesis.

If there are not enough studies, we will use a synthetic effect size (the average) in order to avoid dependence between effect sizes. This method provides an unbiased estimate of the mean effect size parameter but overestimates the standard error. Random effects models applied when synthetic effect sizes are involved actually perform better in terms of standard errors than do fixed effects models (L. V. Hedges, 2007). However, tests of heterogeneity when synthetic effect sizes are included are rejected less often than nominal.

If pooling is not appropriate (e.g., the multiple interventions and/or control groups include the same individuals), only one intervention group will be coded and compared to the control group to avoid overlapping samples. The choice of which estimate to include will be based on our risk of bias assessment. We will choose the estimate that we judge to have the least risk of bias (primarily confounding bias) and in case of equal scoring, the missing outcome data domain will be used).

##### Multiple studies using the same sample of data

In some cases, several studies may have used the same sample of data or some studies may have used only a subset of a sample used in another study. We will review all such studies, but in the meta‐analysis we will only include one estimate of the effect from each sample of data. This will be done to avoid dependencies between the “observations” (i.e., the estimates of the effect) in the meta‐analysis. The choice of which estimate to include will be based on our risk of bias assessment of the studies and the sample size. We will choose the estimate from the study that we judge to have the least risk of bias (primarily confounding bias). However, if the studies are rated equally on every risk of bias item, we will include the study using the largest share/full set of participants in the data synthesis.

##### Multiple time points

When the results are measured at multiple time points, each outcome at each time point will be analysed in a separate meta‐analysis with other comparable studies taking measurements at a similar time point. As a general guideline, these will be grouped together as follows: (1) 0–1 year follow up, (2) 1–2 year follow up, and (3) more than 2 year follow up. However, should the studies provide viable reasons for an adjusted choice of relevant and meaningful duration intervals for the analysis of outcomes, we will adjust the grouping.

#### 3.3.7 Dealing with missing data

Missing data in the individual studies will be assessed using the risk of bias tool. Studies must permit calculation of a numeric effect size for the outcomes to be eligible for inclusion in the meta‐analysis. Where studies have missing summary data, such as missing standard deviations, we will derive these where possible from, for example, *F* ratios, *t* values, *χ* ^2^ values, and correlation coefficients using the methods suggested by Lipsey and Wilson (2001). If these statistics are also missing, the review authors will request information from the study investigators.

If missing summary data necessary for the calculation of effect sizes cannot be derived or retrieved, the study results will be reported in as much detail as possible, that is, the study will be included in the review but excluded from the meta‐analysis.

#### 3.3.8 Assessment of heterogeneity

Heterogeneity among primary outcome studies will be assessed with *χ* ^2^ (*Q*) test, and the *I* ^2^, and *τ* ^2^ statistics (Higgins et al., 2003). Any interpretation of the *χ* ^2^ test will be made cautiously on account of its low statistical power.

#### 3.3.9 Assessment of reporting biases

Reporting bias refers to both publication bias and selective reporting of outcome data and results. Here, we state how we will assess publication bias.

We will use funnel plots for information about possible publication bias if we find sufficient studies (Higgins & Green, 2011). However, asymmetric funnel plots are not necessarily caused by publication bias (and publication bias does not necessarily cause asymmetry in a funnel plot).

In general, asymmetry is a sign of small‐study effects, of which there can be many causes beside publication bias (Sterne et al., 2005).

Instead of trying to interpret the funnel plots as direct evidence of publication bias, or the lack thereof, we will perform sensitivity analyses for publication bias in meta‐analyses as suggested by Mathur and VanderWeele (2020). This method gives a value of how large ratios of publication probabilities (that is the likelihood of affirmative results to be published relative to non‐affirmative results) would have to be to alter the results and therefore indicate how robust the meta‐analysis is to publication bias.

#### 3.3.10 Data synthesis

The proposed project will follow standard procedures for conducting systematic reviews using meta‐analysis techniques.

The overall data synthesis will be conducted where effect sizes are available or can be calculated, and where studies are similar in terms of the outcome measured. Meta‐analysis of outcomes will be conducted on each metric separately (as outlined in Section 4.1.4).

As different computational methods may produce effect sizes that are not comparable, we will be transparent about all methods used in the primary studies (research design and statistical analysis strategies) and use caution when synthesising effect sizes. Special caution will be taken concerning studies using regression discontinuity (RD) to estimate a local average treatment effect (LATE) (Angrist & Pischke, 2009). These will be included, but may be subject to a separate analysis depending on the comparability between the LATE's and the effects from other studies. We will in any case check the sensitivity of our results to the inclusion of RD studies. In addition, we will discuss the limitation in generalisation of results obtained from these types of studies.

When the effect sizes used in the data synthesis are odds ratios, they will be log transformed before being analysed. The reason is that ratio summary statistics all have the common feature that the lowest value that they can take is 0, that the value 1 corresponds with no intervention effect, and the highest value that an odds ratio can ever take is infinity. This number scale is not symmetric. The log transformation makes the scale symmetric: the log of 0 is minus infinity, the log of 1 is zero, and the log of infinity is infinity.

Studies that have been coded with a Critical risk of bias will not be included in the data synthesis.

As the intervention deals with diverse populations of participants (from different countries with different disabilities or special educational needs), and we therefore expect heterogeneity among primary study outcomes, all analyses of the overall effect will be inverse variance weighted using random effects statistical models that incorporate both the sampling variance and between study variance components into the study level weights. Random effects weighted mean effect sizes will be calculated using 95% confidence intervals and we will provide a graphical display (forest plot) of effect sizes. Graphical displays for meta‐analysis performed on ratio scales sometimes use a log scale, as the confidence intervals then appear symmetric. This is however not the case for the software Revman 5 which we plan to use in this review.^3^ The graphical displays using odds ratios and the mean effect size will be reported as an odds ratio.

For subsequent analyses of moderator variables (gender, age, type of special need, coteaching versus only general education teachers, full vs. part time inclusion) that may contribute to systematic variations, we will use the mixed‐effects regression model. This model is appropriate if a predictor explaining some between‐studies variation is available but there is a need to account for the remaining uncertainty (Hedges & Pigott, 2004; Konstantopoulos, 2006).

We expect that several studies have used the same sample (or sub samples) of data. We will review all such studies, but in the meta‐analysis we will only include one estimate of the effect from each sample of data. This will be done to avoid dependencies between the “observations” (i.e., the estimates of the effect) in the meta‐analysis. The choice of which estimate to include will be based on our quality assessment of the studies. We will choose the estimate from the study that we judge to have the least risk of bias, with particular attention paid to confounding bias. If two (or more) studies uses the same (sub) samples and are rated equally on every risk of bias item, we will include the study using the largest share/full set of participants in the data synthesis.

We anticipate that several studies provide results separated by for example age and/or gender. We will include results for all age and gender groups. To take into account the dependence between such multiple effect sizes from the same study, we will apply the robust variance estimation (RVE) approach (Hedges et al., 2010). An important feature of this analysis is that the results are valid regardless of the weights used. For efficiency purposes, we will calculate the weights using a method proposed by Hedges et al. (2010). This method assumes a simple random‐effects model in which study average effect sizes vary across studies (*τ* ^2^) and the effect sizes within each study are equi correlated (*ρ*). The method is approximately efficient, since it uses approximate inverse‐variance weights: they are approximate given that *ρ* is, in fact, unknown and the correlation structure may be more complex. We will calculate weights using estimates of *τ* ^2^, setting *ρ* = 0.80 and conduct sensitivity tests using a variety of *ρ* values to asses if the general results and estimates of the heterogeneity are robust to the choice of *ρ*. We will use the small sample adjustment to the residuals used in RVE as proposed by Bell and McCaffrey (2002) and extended by McCaffrey et al. (2001) and by Tipton (2015). We will use the Satterthwaite degrees of freedom (Satterthwaite, 1946) for tests as proposed by Bell and McCaffrey (2002) and extended by Tipton (2015). We will use the guidelines provided in Tanner‐Smith and Tipton (2014) to evaluate if there are enough studies for this method to consistently estimate the standard errors.

If there is not a sufficient number of studies to use RVE, we will conduct a data synthesis where we use a synthetic effect size (the average) in order to avoid dependence between effect sizes.

#### 3.3.11 Subgroup analysis and investigation of heterogeneity

We will investigate the following factors with the aim of explaining potential observed heterogeneity: study‐level summaries of participant characteristics (e.g., studies considering a specific disability category such as “learning disorders” or “students with autism spectrum disorders”; a specific gender or age group or studies where separate effects for girls/boys or age groups (e.g., 6–10/11–16/17–19 year old) are available. Furthermore, we will explore the specific characteristics of the inclusive educational setting as outlined in the section: *The intervention* (e.g., full‐time or part‐time inclusion and involvement or not involvement of additional teaching and/or pedagogical resources). If the number of included studies is sufficient and given there is variation in the covariates (disability category, age, gender and characteristics of the inclusive educational setting), we will perform moderator analyses (multiple meta‐regression using the mixed model) to explore how observed variables are related to heterogeneity.

If there is a sufficient number of studies, we will apply the RVE approach and use approximately inverse variance weights calculated using a method proposed by Hedges et al. (2010). This technique calculates standard errors using an empirical estimate of the variance: it does not require any assumptions regarding the distribution of the effect size estimates. The assumptions that are required to meet the regularity conditions are minimal and generally met in practice. This more robust technique is beneficial because it takes into account the possible correlation between effect sizes separated by the covariates within the same study (e.g., age or gender separated effects) and allows all of the effect size estimates to be included in meta‐regression. We will calculate weights using estimates of *τ* ^2^, setting *ρ* = 0.80 and conduct sensitivity tests using a variety of *ρ* values to asses if the general results are robust to the choice of *ρ*. We will use the small sample adjustment to the residuals used in RVE and the Satterthwaite degrees of freedom (Satterthwaite, 1946) for tests (Tipton, 2015). The results in Tipton (2015) suggest that the degrees of freedom depend on not only the number of studies but also on the type of covariates included in the meta‐regression. The degrees of freedom can be small, even when the number of studies is large if a covariate is highly unbalanced or a covariate with very high leverage is included. The degrees of freedom will vary from coefficient to coefficient. The corrections to the degrees of freedom enable us to assess when the RVE method performs well. As suggested by Tanner‐Smith and Tipton (2014) and Tipton (2015), if the degrees of freedom are smaller than four, the RVE results should not be trusted.

We will report 95% confidence intervals for regression parameters. We will estimate the correlations between the covariates and consider the possibility of confounding. Conclusions from meta‐regression analysis will be cautiously drawn and will not solely be based on significance tests. The magnitude of the coefficients and width of the confidence intervals will be taken into account as well. Otherwise, single factor subgroup analysis will be performed. The assessment of any difference between subgroups will be based on 95% confidence intervals. Interpretation of relationships will be cautious, as they are based on subdivision of studies and indirect comparisons.

In general, the strength of inference regarding differences in treatment effects among subgroups is controversial. However, making inferences about different effect sizes among subgroups on the basis of between‐study differences entails a higher risk compared to inferences made on the basis of within study differences; see Oxman and Guyatt (1992). We will therefore use within study differences where possible.

We will also consider the degree of consistency of differences, as making inferences about different effect sizes among subgroups entails a higher risk when the difference is not consistent within the studies (see Oxman & Guyatt, 1992).

#### 3.3.12 Sensitivity analysis

Sensitivity analysis will be carried out by restricting the meta‐analysis to a subset of all studies included in the original meta‐analysis and will be used to evaluate whether the pooled effect sizes are robust across components of risk of bias. We will consider sensitivity analysis for each domain of the risk of bias checklists and restrict the analysis to studies with a low risk of bias.

Sensitivity analyses with regard to research design and statistical analysis strategies in the primary studies will be an important element of the analysis to ensure that different methods produce consistent results.

##### Treatment of qualitative research

We do not plan to include qualitative research.

## CONTRIBUTIONS OF AUTHORS

- *Content*:
  Nina Thorup Dalgaard is a psychologist, PhD Nina has previously worked as both an educational psychologist within a primary school setting and as a clinical child psychologist and thus has knowledge about the socioemotional and cognitive development of children.
  Anja Bondebjerg holds a Master's degree in Sociology and has worked extensively with systematic reviews and research mappings in the fields of education and early childhood education and care. She is knowledgeable regarding the structure and process of conducting systematic reviews.
- *Systematic review methods*:
  Trine Filges, PhD (economics): is an experienced systematic reviewer and methodologist, having completed a number of systematic reviews in social welfare topic areas as well as in the field of education. Trine has published thirteen Campbell Systematic reviews, is currently the lead reviewer on three Campbell Systematic Reviews, further involved as a reviewer in two Campbell Systematic Reviews and has published systematic and meta‐analytic reviews in high‐impact journals. Trine's fields of expertise are systematic review methods and statistical analysis; and she will contribute to the quantitative data extraction, methodological quality appraisal and meta‐analysis.

Bjørn C. A. Viinholt (information specialist) has 4 years of experience in developing and writing systematic reviews. As a part of undertaking systematic reviews, Bjørn has experience in developing systematic search strategies and processes of reference management. Bjørn will contribute with assisting and development of the systematic search strategy, executing the searches, and assist with reference management and grey literature searches. Bjørn will also assist with aspects relating to systematic literature searches in Campbell review methodology.

## Footnotes

1 The review was retrieved 2.10.2019 from: https://files.eric.ed.gov/fulltext/ED519296.pdf.

2 For more information on the IDEA Act disability categories, go to: https://sites.ed.gov/idea/regs/b/a/300.8 (the U.S. Department of Education's Individuals with Disabilities Education Act (IDEA) website).

3 If we apply robust variance estimation, the analysis will be conducted in STATA or R as robust variance estimation is not implemented in Revman 5.

## APPENDIX A FIRST AND SECOND LEVEL SCREENING

First level screening is on the basis of titles and abstracts. Second level is on the basis of full text

Reference id. No.:

Reviewers initials:

Source:

Year of publication:

Country/countries of origin:

Author(s):

The study will be excluded if one or more of the answers to Questions 1–4 are “No.” If the answers to Questions 1 to 4 are “Yes” or “Uncertain,” then the full text of the study will be retrieved for second level eligibility. All unanswered questions need to be posed again on the basis of the full text. If not enough information is available, or if the study is unclear, the author of the study will be contacted if possible.

**Screening questions:**

1. Does the study measure the effects of inclusion (may be referred to as inclusive education, mainstreaming, integrated placement, cross‐categorical instruction or coteaching)?
  Yes ‐ include
  No – if no then stop here and exclude
  Uncertain ‐ include
  Question 1 guidance:
  *Inclusion* refers to an educational setting with a mixture of children with and without special educational needs. In the present review, the intervention termed *inclusion* may thus be defined as any given group composition within a general educational setting which contains at least one child with an identified special educational need. Placement in an inclusive setting may be full time or part time.The ratio of students with and without special needs may vary. The general education teachers may be provided with support and continuing professional development aimed at helping the teachers accommodate the needs of special education students. Special education teachers may also be present within the general education setting or there may be teaching assistants present. It is often referred to as coteaching when two or more professionals deliver substantive instruction to a diverse or blended group of students within the same physical space.
2. Does the study measure effects for students with special needs?
  Yes—include
  No—if no then stop here and exclude
  Uncertain—include
  Question 2 guidance:
  The population of this review are special needs children in grades K to 12 (or the equivalent in European countries) in special education. The review will include special needs children in grades K to 12 (or the equivalent in European countries) in special education. Studies that meet inclusion criteria will be accepted from all countries.In this review we apply the widely used definition from the US Individuals with Disabilities Education Act (IDEA), in which special needs are divided into 13 different disability categories under which children are eligible for services[1]. These categories are:
  - specific learning disability
  - other health impairment
  - autism spectrum disorder
  - emotional disturbance
  - speech or language impairment
  - visual impairment
  - deafness
  - hearing impairment
  - deaf‐blindness
  - orthopaedic impairment
  - intellectual disability
  - traumatic brain injury
  - multiple disabilities
  Studies focusing exclusively on children without special educational needs will not be eligible.
3. Is the report/article a quantitative study with a comparison condition?

Yes—include

No—if no then stop here and exclude

Uncertain—include

Question 4 guidance:

We are only interested in primary quantitative studies with a comparison group. Eligible study designs are: Randomised controlled trials (RCTs), Quasi‐randomised controlled trial designs (QRCTs), Quasi‐experimental studies (QES) and repeated‐measures experimental designs in which the same caregiver and/or children are observed under different conditions within a short time span. Studies reporting associations in cohort, cross‐sectional and longitudinal study designs without a comparison group are not eligible.

We are not interested in theoretical papers on the topic or surveys/reviews of studies of the topic. (This question may be difficult to answer on the base of titles and abstracts alone.).

## APPENDIX B DATA EXTRACTION

**Table:**

**Names of author(s)**
**Title**
**Language**
**Journal**
**Year**
**Country**
**Type of school setting (including grade level)**
**Programme feature:** *Study design*, (brief description)
**Programme feature:** *Intervention* (type of inclusive setting such as full or part time)
**Programme feature** *Outcomes*: (academic achievement, socioemotional or wellbeing)
**Programme feature** *Participants* (type of special needs/disability category, gender)
**Programme feature** *teacher characteristics*, (number of teachers, educational background, years of experience, continuous professional development)
**Type of data used in study (independent observation, questionnaire, other (specify))**
**Level of aggregation (individual and/or setting)**
**Time period covered by analysis (divide into intervention and follow up)**
**Sample size (divide into treated/comparison)**

## APPENDIX C OUTCOME MEASURES

**Out come data**

**Dichotomous outcome data**

**Table:**

OUTCOME	TIME POINT (s) (record exact time from participation, there may be more than one, record them all)	SOURCE	VALID Ns	CASES	NON‐CASES	STATISTICS	Pg. # & NOTES
		Questionnaire Admin data Other (specify) Unclear	Participation Comparison	Participation Comparison	Participation Comparison	RR (risk ratio) OR (odds ratio) SE (standard error) 95% CI DF *P*‐value (enter exact *p* value if available) Chi2 Other	

OUTCOME	TIME POINT (s) (record exact time from participation, there may be more than one, record them all)	SOURCE (specify)	VALID Ns	Means	SDs	STATISTICS	Pg. # & NOTES
		Questionnaire Admin data Other (specify) Unclear	Participation Comparison	Participation Comparison	Participation Comparison	P t F Df ES Other	

*Repeat as need

## APPENDIX D ASSESSMENT OF RISK OF BIAS IN INCLUDED STUDIES

### C.1 | User guide for unobservables

Systematic baseline differences between groups can compromise comparability between groups. Baseline differences can be observable (e.g., age and gender) and unobservable (to the researcher; e.g., motivation and “ability”). There is no single non‐randomised study design that always solves the selection problem. Different designs solve the selection problem under different assumptions and require different types of data. Especially how different designs deal with selection on unobservables varies. The “right” method depends on the model generating participation, that is, assumptions about the nature of the process by which participants are selected into a programme.

As there is no universal correct way to construct counterfactuals we will assess the extent to which the identifying assumptions (the assumption that makes it possible to identify the counterfactual) are explained and discussed (preferably the authors should make an effort to justify their choice of method). We will look for evidence that authors using for example (this is NOT an exhaustive list):

**Natural experiments:**

Discuss whether they face a truly random allocation of participants and that there is no change of behaviour in anticipation of, for example, policy rules.

**Matching (including propensity scores):**

Explain and discuss the assumption that there is no selection on unobservables, only selection on observables.

**(Multivariate, multiple) Regression:**

Explain and discuss the assumption that there is no selection on unobservables, only selection on observables. Further discuss the extent to which they compare comparable people.

**Regression Discontinuity:**

Explain and discuss the assumption that there is a (strict!) RD treatment rule. It must not be changeable by the agent in an effort to obtain or avoid treatment. Continuity in the expected impact at the discontinuity is required.

**Difference‐in‐difference (Treatment‐control‐before‐after):**

Explain and discuss the assumption that the trends in treatment and control groups would have been parallel, had the treatment not occurred.

### C.2 | Justification of exclusion of studies using an instrumental variable (IV) approach

Studies using instrument variables (IV) for causal inference in non‐randomised studies will not be included as the interpretation of IV estimates is challenging. IV only provides an estimate for a specific group namely, people whose behaviour change due to changes in the particular instrument used. It is not informative about effects on never‐takers and always‐takers because the instrument does not affect their treatment status. The estimated effect is thus applicable only to the subpopulation whose treatment status is affected by the instrument. As a consequence, the effects differ for different IVs and care has to be taken as to whether they provide useful information. The effect is interesting when the instrument it is based on is interesting in the sense that it corresponds to a policy instrument of interest. Further, if those that are affected by the instrument are not affected in the same way the IV estimate is an average of the impacts of changing treatment status in both directions, and cannot be interpreted as a treatment effect. To turn the IV estimate into a LATE requires a monotonicity assumption. The movements induced by the instrument go in one direction only, from no treatment to treatment. The IV estimate, interpreted as a LATE, is only applicable to the complier population, those that are affected by the instrument in the “right way.” It is not possible to characterise the complier population as an observation's subpopulation cannot be determined and defiers do not exist by assumption.

In the binary‐treatment–binary‐instrument context, the IV estimate can, given monotonicity, be interpreted as a LATE; that is, the average treatment effect for the subpopulation of compliers. If treatment or instruments are not binary, interpretation becomes more complicated. In the binary‐treatment–multivalued‐instrument (ordered to take values from 0 to *J*) context, the IV estimate, given monotonicity, is a weighted average of pairwise LATE parameters (comparing subgroup *j* with subgroup *j* − 1). The IV estimate can thus be interpreted as the weighted average of average treatment effects in each of the *J* subgroups of compliers. In the multivalued‐treatment (ordered to take values from 0 to *T*)—multivalued‐instrument (ordered to take values from 0 to *J*) context, the IV estimate for *each pair of instrument values*, given monotonicity, is a weighted average of the effects from going from *t*‐1 to *t* for persons induced by the change in the value of the instrument to move from any level below *t* to the level *t* or any level above. Persons can be counted multiple times in forming the weights.

Based on Angrist and Pischke (2009), Heckman and Urzúa (2010) and Heckman et al. (2006).
